# The Effect of Immunonutrition on the Postoperative Complications in Thymoma with Myasthenia Gravis

**DOI:** 10.1155/2016/8781740

**Published:** 2016-11-10

**Authors:** Yanzhong Xin, Hongfei Cai, Lihui Wu, Youbin Cui

**Affiliations:** ^1^Department of Thoracic Surgery, The First Hospital of Jilin University, 71 Xinmin Street, Changchun 130021, China; ^2^Institute for the Control of Agrochemicals, Ministry of Agriculture, Beijing 100125, China

## Abstract

*Object*. To test whether preoperative immunonutrition is efficacious in reducing postoperative complications in patients of thymoma with myasthenia gravis (MG).* Material and Methods*. A total of 244 patients operated on for thymoma with myasthenia gravis were prospectively assigned to two groups, each receiving seven-day preoperative and seven-day postoperative nutrition. The patients in immunonutrition group were given oral immunonutrition (IN). The patients in control group received oral standard nutrition. Immunonutritional and inflammatory biomarkers (IgA, IgG, IgM, CD3t, CD4t, CD8t, CD4t/CD8t ratio, NK-cell, prealbumin, albumin, white blood cells counts, and C-reactive protein) and clinical variables (age, gender, BMI, performance status, type of thymoma, type of MG, operative time, pathology, operative approach, postoperative complications, quantity of drainage, hospital stays) were examined.* Results*. A significant reduction in the length of hospital stay, quantity of drainage, and postoperative complications was observed in the IN group (*p* < 0.05). An increase in the level of IgA, IgG, IgM, CD3+T, CD4+T, CD4+T/CD8+T, WBC, CRP, and NK-cell in the IN group was observed after thymectomy, while a decrease was seen with regard to prealbumin and albumin (*p* < 0.05).* Conclusion*. Preoperative immunonutrition support is effective in reducing postoperative complications in patients of thymoma with MG. It helps to lower the risk of postoperative infectious complications and hospital stays.

## 1. Introduction

Thymoma is a rare disease but accounts for about 50% of tumors in the anterior mediastinum [1, 2]. Thymectomy is the golden standard for the treatment of thymoma due to its malignant potential [3–5]. Considering its immunological characteristics, a lot of scholars have recommended performing en bloc resection of thymic cells, because this is an important prognostic factor in the treatment of thymoma [6, 7]. It is self-evident that complications after operation are unavoidable, which mainly include pneumonia, infection of incision, and respiratory insufficiency [8]. Besides, thymoma usually is associated with many autoimmune diseases, including systemic lupus erythematosus (SLE), polymyositis, Good's syndrome, pure red cell aplasia (PRCA), and myasthenia gravis (MG) which is the most common symptom complicated with the thymoma [9]. Although approximately 50% of the patients have a chance of drug- and symptom-free life after the operation [10, 11], the morbidity and mortality could be increased during postoperation [12], and many of them are the independent factors affecting the prognosis, especially the MG. If postthymectomy myasthenic crisis occurred, it can make the pneumonia, which is a common complication, life-threatening [13]. But it is difficult to predict when such complications will come up after the operation, and by now, there has been no effective approach to preventing these complications or reducing the incidence.

Immunonutrition (IN), which is the combination of standard nutritional formulas and immunonutrients, has been widely exploited recently. The immunonutrients are glutamine, arginine, and polyunsaturated fatty acids (omega-3), among others, which can improve the immunity and nutrition effectively. They can modulate inflammatory responses and enhance protein synthesis and then increase immune responses. It has been proved that the perioperative application of immunonutrition is an effective and more prevailing therapeutic strategy [14, 15]. As they can reduce infectious and other postoperative complications, they have been extensively applied in many kinds of disease and status, especially in digestive tumor and malnourished patients [16–19]. Strong evidence, including some randomized, double-blind trials, demonstrates that perioperative immunonutrition support significantly reduced the incidence of pneumonia and hospital stay [20]. Meanwhile, some studies reported that the treatment of immunoglobulin is effective for the MG crisis and severe MG. In some cases, intravenous immunoglobulin could be an alternative approach when therapeutic plasma exchange is unsuitable because of its high possibility of developing cardiopulmonary failure [21]. Some other studies reported that improving the nutrition and immunity rapidly is beneficial for the inflammation [22]. But it is still unknown whether or not immunonutrition is effective in reducing the morbidity and mortality in the perioperative period of thymectomy.

As such, the object of this study was to examine whether preoperative immunonutrition is efficacious in reducing postoperative complications in patients of thymoma with MG.

## 2. Materials and Methods

### 2.1. Patients

A prospective, nonrandomized, interventional, single-blind cohort study was designed. 244 well-nourished patients of thymoma with MG from January 2012 to December 2015 were enrolled. No patient received preoperative chemoradiotherapy. Informed consent was obtained from all the patients.

### 2.2. Feeding Regimens

The oral immunonutrition (IN) supplement group receiving immune-enriched diet was compared with the oral standard nutrition (SN) supplement group receiving the standard nutrition diet, which started 7 days prior to thymectomy and lasted 7 days postoperatively with the parenteral alimentation on the day of surgery. The ingredients of formula are listed in Table 1.

We performed blood test on postoperative 1st, 3rd, 5th, and 7th days and preoperative first day, respectively. Outcome measures included clinical variables (age, gender, BMI, performance status, type of thymoma, type of MG, operative time, pathology, operative approach, postoperative complications, quantity of drainage, and hospital stays) and immunonutritional and inflammatory biomarkers (IgA, IgG, IgM, CD3t, CD4t, CD8t, CD4t/CD8t ratio, NK-cell, prealbumin, albumin, white blood cells counts, and C-reactive protein).

### 2.3. Statistical Analyses

We performed statistical comparison using Chi squared test, Student's *t*-test, and Fisher's exact test for the comparison. It was considered statistically significance at *p* < 0.05. Statistics analysis was performed using SPSS v 18.00.

## 3. Results

102 (41.8%) subjects under investigation received the immunonutrition support, and 142 (58.2%) patients did not. Concerning baseline characteristics between the two groups, no significant difference was observed with respect to age, gender, BMI, performance status, type of thymoma, type of MG, operative time, pathology, or operative approach. Comparatively a significant decrease in the duration of hospital stay, quantity of drainage, and postoperative complications was observed in the IN group (*p* < 0.05) (Table 2).

**Figure 1:**
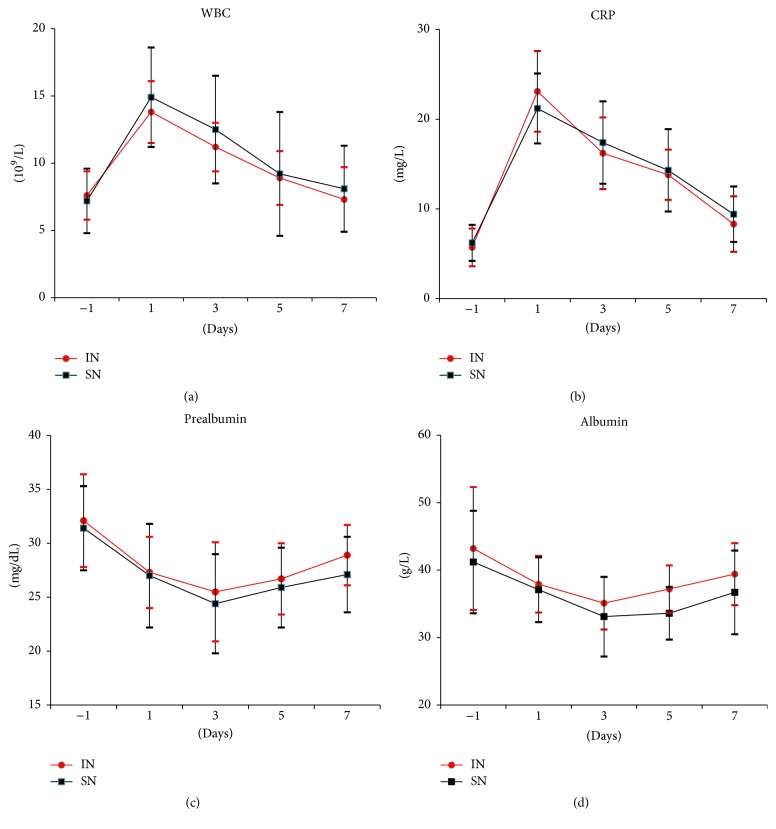


As shown in Table 3, 56 (23.0%) patients experienced postoperative complications for the entirety, and 21 (20.6%) of the 102 patients did in IN group (*p* < 0.05). Pneumonia and myasthenic crisis remain the most common postoperative complications; with regard to pneumonia, the incidence of pneumonia in the IN group was significantly lower than in the control group (*p* < 0.05). The IN group was inclined to reduce the total infectious complications rate (*p* < 0.05). The significant relativity between fast reconversion of WBC, CRP, prealbumin, albumin, and immunonutrition support was observed through the analysis of subgroups with infectious complications (Figures 1(a), 1(b), 1(c), and 1(d)).

On the other hand, decreased hospital stays, length of extubation, intensive care unit stay, and pneumonia with the IN group were observed significantly through the analysis of subgroups with myasthenic crisis (Table 4).

The preoperative inflammatory and immune markers including IgA, IgG, IgM, CD3+T, CD4+T, CD8+T, CD4+T/CD8+T, NK-cell, prealbumin, albumin, WBC, and CRP between the two groups were not statistically different. On the first postoperative day, no significant difference was observed in the mentioned markers between the two groups. However, on the third postoperative day, the levels of WBC, CRP, and NK-cell dramatically decreased in the IN group (*p* < 0.05). On the fifth day, in contrast to the significant decrease in the IN group concerning the level of WBC, CRP, and NK-cell, an increase was seen in the level of prealbumin and albumin (*p* < 0.05). Although CD8+T showed no significant differences, an increase in the level of IgA, IgG, IgM, CD3+T, CD4+T, and CD4+T/CD8+T in the IN group was observed on the seventh day, the same as the value of prealbumin and albumin. The level of WBC, CRP, and NK-cell was significantly lower than that in SN group (Table 5).

## 4. Discussion

Thymectomy is the most common tumor of anterior mediastinum, and 30–50% of the thymoma patients have myasthenia gravis [1]. About 15–20% of MG patients will develop myasthenic crisis, which is a life-threatening emergency always caused by infections [23–25]. Thymectomy may reduce the morbidity of this status [26], while thymectomy was always accompanied with high postoperative complications, especially infectious complications including pneumonia, which limited its efficacy. Given that immunosuppression caused by surgical stress is one of the most important factors in developing complications [27], the effects of immunonutrition on outcome after major operation for cancer or after severe injury have been frequently reported; however, the effects for thymoma have never been mentioned. Therefore we designed this study to examine the effect of immunonutrition on the postoperative complications in thymoma with myasthenia gravis.

Immunonutrition can not only modulate inflammatory responses and enhance protein synthesis and then increase immune responses, but also promote the control of the immune obstacle during the early postoperative phase and improve intestinal blood supply and oxygen metabolism [28–31]. Lots of studies have demonstrated that perioperative immunonutrition support significantly reduced the incidence of infectious complications. In their research for patients undergoing major abdominal cancer surgery, Giger et al. concluded that perioperative immunonutrition support significantly reduces inflammation and postoperative complications compared with postoperative diet administration alone [18]. In addition, Rowan and his collaborators published their results that perioperative immunonutrition may lead to significant reductions of postoperative complications in high-risk head and neck cancer patients [32]. Moreover, Silvestri et al. compared the clinical characteristics of patients after pancreaticoduodenectomy between immunonutrition group and control group and then obtained the result that immunonutrition helps to lower the risk of postoperative infectious complications and of hospital stays [33–35]. In our cohort, we obtained similar results with patients of thymoma with MG; the morbidity of postoperative complications, especially infectious complications, was significantly reduced in IN group.

Although some studies showed no significant difference with hospital stay in immunonutrition group [33, 36], different outcome was observed in our study. We attribute the result to the choice of the subjects. Moya and his team studied the effect of immunonutrition on the patients under colorectal resection in an enhanced recovery after surgery (ERAS) protocol and drew a conclusion that the average postoperative hospital stay was not significantly different between the two groups. Their median postoperative hospital stay, which was only 5 days, maybe interfered by the applications of ERAS [33]. Song and his collaborators reviewed 9 studies involving 785 patients undergoing gastric resection concerning enteral immunonutrition and obtained a result that length of hospitalization was not improved, but they analysed that their outcome with heterogeneity caused by different compositions, different timing of administration of immunonutrient, insufficient quantity of sample size, and number of eligible researches in most of sensitive analyses with subgroup analysis may impair their conclusions [36]. We surmised that it is the heterogeneity that contributes to the different results.

In the analysis of the subgroup of myasthenic crisis, the hospital stay, length of extubation, and ICU stay were significantly lower in IN group than those in SN group. The patients with myasthenic crisis were inclined to be accompanied by high morbidity of pneumonia, especially ventilator associated pneumonia. Rabinstein and Mueller-Kronast reported that pneumonia was significantly associated with extubation failure, which considerably prolonged intensive care unit stay and hospital stay [37]. In our research, there were more patients with myasthenic crisis in the SN group, which may contribute to the longer hospital stay, extubation, and ICU stay. It is assumed that the different morbidity of pneumonia may contribute to the hyperdispersion of the subjects investigated. Immunonutrition provides a new option for improving the prognosis of our patients.

Song and his collaborators conducted a meta-analysis on the patients from when they underwent surgery for gastric cancer to enteral immunonutrition. They came to the conclusion that the immune markers increased in immunonutrition group [36]. Shimogawa et al. reported that the inflammatory markers including CRP and WBC count were decreased in the IN group of patients with severe intracranial hemorrhage during the acute stage [38]. In our study, the increased immune markers were observed five days postoperatively and the decreased inflammatory markers were observed to begin on the third postoperative day. Although there was still significant difference between the two groups on the seventh postoperative day, there has been no clinical significance because the value of CRP and WBC count has restored to normal in both groups. With the analysis of the subgroup of infectious complications, there was significant difference with the reconversion of WBC count, CRP, prealbumin, and albumin between the two groups. The morbidity of myasthenic crisis was lower in IN group.

Calder reported that *ω*-3 polyunsaturated fatty acids (*ω*-3 PUFAs), as a main component of the immune nutrients, ensured the maintenance of membrane fluidity and the sufficient function of membrane proteins. Its dysbolism maybe leads to many human diseases including immune disorders or cancers [39]. Furthermore, it demonstrates strong anti-inflammatory and immunomodulatory effects via influencing the synthesis of eicosanoid mediators prostaglandins (PGs), thromboxanes (TXs), and leukotrienes (LTs). In addition to this, its production stimulated peripheral monocytes [40]. Tossou et al. reported that tryptophan played the role of regulating physiological functions and intestinal permeability in the alimentary tract [41]. Hyeyoung Kim reviewed the glutamine, another essential immune nutrient. He pointed out that glutamine always played an essential role in nitrogen transport in vivo and served as a substrate for renal ammoniagenesis. Moreover, it influenced the expression of heat shock proteins and stimulates nucleotide synthesis and activates signaling mediators [42–44]. Arginine, as the important component part of immune nutriment, stimulates anabolic hormone release, improves nitrogen balance, and has immunostimulatory and thymotrophic functions. It also plays roles in nitrogen metabolism, creatine, and polyamine synthesis [45]. On the whole, immunonutrition, including *ω*-3 PUFAs, glutamine, and arginine, could modulate inflammatory responses, enhance nitrogen balance and protein synthesis, increase host immune responses, and then shorten the course of disease, reduce postoperative complications, and improve the prognosis of patients.

Interestingly, there is also significant difference between quantity of drainage and IN group. We speculate that this result may contribute to the fast reconversion of the albumin and prealbumin because it was proven that preoperative immunonutrition could provide a significant increase in prealbumin levels [46]. Xu et al. compared the effects on nutritional status between immunonutrition pharmaceutics group and standard nutrition group and reached a conclusion that higher IgG, CD4/CD8 ratio, and prealbumin were significantly relevant with immunonutrition support [47].

Though we exclude the malnutrition subjects so as to reduce the bias as much as possible, some limitation still exists in our study. For one thing, distribution of characteristics of subjects restricted by the single centre may be different from general regularity. For another thing, the sample size for the subgroups is not big enough.

## 5. Conclusion

Preoperative immunonutrition support is effective in reducing postoperative complications in patients of thymoma with MG. It helps to lower the risk of postoperative infectious complications and hospital stays.

## Figures and Tables

**Table 1:** Composition of immunonutrition.

Composition (per 100 ml)	Amount (g)
Proteins, g	7.6
L-arginine, g	1.0
Ribonucleic Acid, g	0.2
Fats (total), g	3.9
Saturated fatty acids	1.8
Medium chain triglycerides	1.12
Monounsaturated	0.73
Polyunsaturated	1.3
Linoleic acid	0.6
U-3 fatty acids	0.77
Carbohydrates, g	13.3
Sucrose, g	10.5
Lactose, g	<0.02
Soluble fibers, g	1.4
Energy, kcal	141
Iron, mg	1.7
Zinc, mg	2.2
Copper, *μ*g	169
Vitamin A, *μ*g	200
Vitamin D, *μ*g	3.3
Vitamin E, mg	4.5
Vitamin B1, mg	0.33
Vitamin B2, *μ*g	0.33
Vitamin B6, *μ*g	0.33
Vitamin B12, *μ*g	1
Vitamin C, mg	33
Water, g	77

**Table 2:** Patient characteristics between two groups.

	Immunonutrition (*n* = 102)	Standard nutrition (*n* = 142)	*p*
Age	52.3 ± 12.4	53.2 ± 13.1	>0.05
Gender			>0.05
Male	48 (47.1%)	67 (47.2%)	
Female	54 (52.9)	75 (52.8%)	
BMI	22.8 ± 4.1	23.0 ± 4.0	>0.05
Performance status			>0.05
1	20 (19.6%)	30 (21.1%)	
2	66 (64.7%)	92 (64.8%)	
3	16 (15.7%)	20 (14.1%)	
Type of thymoma			>0.05
I	22 (21.6%)	31 (21.8%)	
II	78 (76.5%)	108 (76.1%)	
III	2 (1.9%)	3 (2.1)	
Type of MG			>0.05
I	22 (21.6%)	33 (23.2%)	
II	63 (61.9)	85 (59.9%)	
III	12 (11.8%)	16 (11.3%)	
IV	5 (4.9%)	8 (5.6%)	
V	0	0	
Operative time (min)	155.6 ± 62.8	159.8 ± 68.2	>0.05
Pathology			>0.05
A	6 (5.9%)	8 (5.6%)	
AB	42 (41.2%)	60 (42.3%)	
B1	14 (13.7%)	19 (13.4%)	
B2	33 (32.4%)	44 (31.0)	
B3	4 (3.9%)	7 (4.9%)	
C	3 (2.9)	4 (2.8%)	
Operative approach			>0.05
Right chest (VATS)	74 (72.5%)	104 (73.2%)	
Sternal (OPEN)	16 (15.7%)	22 (15.5%)	
Subxiphoid (VATS)	12 (11.8%)	16 (11.3%)	
Postoperative complications	21 (20.6%)	35 (24.6%)	<0.05
Quantity of drainage	452.2 ± 112.5	485.3 ± 123.5	<0.05
Hospital stays	16.5 ± 8.4	20.4 ± 9.4	<0.05

**Table 3:** Details of postoperative complications between two groups.

	IN (*n* = 21)	SN (*n* = 35)	*p*
Pneumonia	7 (33.3%)	14 (40%)	<0.05
Mediastinal infection	1 (4.8%)	1 (2.9%)	>0.05
Infection of incision wound	1 (4.8%)	1 (2.9%)	>0.05
Pleural effusions	1 (4.8%)	1 (2.9%)	>0.05
Myasthenic crisis	8 (38.1)	14 (40%)	<0.05
Atrial fibrillation	1 (4.8%)	1 (2.9%)	>0.05
Vocal cord palsy	0	1 (2.9%)	>0.05
Phrenic nerve injury	1 (4.8%)	1 (2.9%)	>0.05
Sternum separation	1 (4.8%)	1 (2.9%)	>0.05

**Table 4:** Comparison of subgroups of postoperative myasthenic crisis between two groups.

	IN (*n* = 8)	SN (*n* = 14)	*p*
Hospital stays	20.5 ± 4.5	22.6 ± 6.7	<0.05
Length of extubation	6.4 ± 4.5	8.5 ± 6.4	<0.05
ICU stay	7.5 ± 4.5	9.8 ± 6.3	<0.05
Quantity of drainage	455.5 ± 112.4	474.4 ± 128.3	>0.05
Pneumonia	4 (50%)	10 (71.4%)	<0.05
Operative time	160.4 ± 68.6	168.5 ± 75.4	>0.05

**Table 5:** Inflammatory and immunobiomarkers between two groups.

	IN					SN				
	D−1	D1	D3	D5	D7	D−1	D1	D3	D5	D7
IgA (mg/ml)	3.5 ± 1.4	2.1 ± 1.4	2.6 ± 1.2	2.9 ± 1.3	3.4 ± 1.2^*∗*^	3.4 ± 1.3	2.0 ± 1.4	2.5 ± 1.3	2.7 ± 1.4	3.2 ± 1.1
IgG (mg/ml)	16.5 ± 6.3	10.2 ± 4.5	11.3 ± 4.6	14.5 ± 4.3	16.3 ± 5.1^*∗*^	16.3 ± 5.7	10.2 ± 4.4	11.0 ± 4.4	13.5 ± 4.8	15.4 ± 4.5
IgM (mg/ml)	1.5 ± 0.4	1.1 ± 0.4	1.2 ± 0.5	1.3 ± 0.4	1.6 ± 0.3^*∗*^	1.5 ± 0.3	1.0 ± 0.3	1.1 ± 0.3	1.2 ± 0.4	1.4 ± 0.4
CD3+T (10^5^/ml)	18.5 ± 8.4	10.6 ± 3.5	12.4 ± 3.5	15.4 ± 3.8	19.2 ± 4.5^*∗*^	17.8 ± 8.3	11.1 ± 3.8	12.3 ± 3.6	15.2 ± 3.6	17.8 ± 4.0
CD4+T (10^5^/ml)	9.5 ± 5.4	5.8 ± 2.8	6.2 ± 3.2	7.5 ± 3.5	9.4 ± 4.8^*∗*^	9.6 ± 4.8	6.0 ± 2.8	6.3 ± 3.6	7.2 ± 3.4	9.4 ± 4.9
CD8+T (10^5^/ml)	8.4 ± 3.5	4.5 ± 3.2	5.3 ± 3.3	6.5 ± 3.2	7.8 ± 3.2	8.6 ± 3.5	4.5 ± 3.3	5.5 ± 3.3	6.4 ± 3.1	7.7 ± 3.1
CD4+T/CD8+T	1.1 ± 0.1	1.6 ± 0.3	1.2 ± 0.2	1.5 ± 0.4	1.4 ± 0.3^*∗*^	1.2 ± 0.2	1.6 ± 0.2	1.5 ± 0.3	1.4 ± 0.3	1.5 ± 0.3
Pre-ALB (mg/dL)	32.1 ± 4.3	27.3 ± 3.3	25.5 ± 4.6	26.7 ± 3.3^*∗*^	28.9 ± 2.8^*∗*^	31.4 ± 3.9	27.0 ± 4.8	24.4 ± 4.6	25.9 ± 3.7	27.1 ± 3.5
ALB (g/L)	43.2 ± 9.1	37.9 ± 4.2	35.1 ± 3.9	37.2 ± 3.5^*∗*^	39.4 ± 4.6^*∗*^	41.2 ± 7.6	37.1 ± 4.8	33.1 ± 5.9	33.6 ± 3.9	36.7 ± 6.2
NK cell (%)	60.5 ± 12.1	80.4 ± 15.4	75.5 ± 13.3^*∗*^	67.6 ± 13.5^*∗*^	62.5 ± 12.1^*∗*^	61.4 ± 11.7	79.3 ± 16.4	75.4 ± 13.2	68.5 ± 13.1	65.4 ± 12.5
WBC counts	7.6 ± 1.8	13.8 ± 2.3	11.2 ± 1.8^*∗*^	8.9 ± 2.0^*∗*^	7.3 ± 2.4^*∗*^	7.2 ± 2.4	14.9 ± 3.7	12.5 ± 4.0	9.2 ± 4.6	8.1 ± 3.2
CRP (mg/l)	5.7 ± 2.1	23.1 ± 4.5	16.2 ± 4.0^*∗*^	13.8 ± 2.8^*∗*^	8.3 ± 3.1^*∗*^	6.2 ± 2.0	21.2 ± 3.9	17.4 ± 4.6	14.3 ± 4.6	9.4 ± 3.1

IN, immunonutrition; D, day; WBC, white blood cell count; CRP, C-reactive protein; Alb, albumin; Pre-Alb, prealbumin; IgA, immunoglobulin A; IgG, immunoglobulin G; IgM, immunoglobulin M; ^*∗*^significantly different compared with SN.

## Abbreviation

MG: Myasthenia gravis
IN: Immunonutrition
BMI: Body mass index
WBC: White blood cell count
CRP: C-reactive protein
Alb: Albumin
Pre-Alb: Prealbumin
IgA: Immunoglobulin A
IgG: Immunoglobulin G
IgM: Immunoglobulin M
NK-cell: Natural killer cell
SLE: Systemic lupus erythematosus
PRCA: Pure red cell aplasia
SN: Standard nutrition
ERAS: Enhanced recovery after surgery
ICU: Intensive care unit
*ω*-3 PUFAs: *ω*-3 polyunsaturated fatty acids
PGs: Prostaglandins
TXs: Thromboxanes
LTs: Leukotrienes.

## References

[1] Fujii Y. (2013). Thymus, thymoma and myasthenia gravis. *Surgery Today*.

[2] Qu Y.-J., Liu G.-B., Shi H.-S., Liao M.-Y., Yang G.-F., Tian Z.-X. (2013). Preoperative CT findings of thymoma are correlated with postoperative Masaoka clinical stage. *Academic Radiology*.

[3] Skeie G. O., Apostolski S., Evoli A. (2010). Guidelines for treatment of autoimmune neuromuscular transmission disorders. *European Journal of Neurology*.

[4] Davenport E., Malthaner R. A. (2008). The role of surgery in the management of thymoma: a systematic review. *Annals of Thoracic Surgery*.

[5] Falkson C. B., Bezjak A., Darling G. (2009). The management of thymoma: a systematic review and practice guideline. *Journal of Thoracic Oncology*.

[6] Detterbeck F., Youssef S., Ruffini E., Okumura M. (2014). A review of prognostic factors in thymic Malignancies. *Zhongguo Fei Ai Za Zhi*.

[7] Murakawa T., Nakajima J., Kohno T. (2000). Results from surgical treatment for thymoma. 43 years of experience. *The Japanese Journal of Thoracic and Cardiovascular Surgery*.

[8] Kas J., Kiss D., Simon V., Svastics E., Major L., Szobor A. (2001). Decade-long experience with surgical therapy of myasthenia gravis: early complications of 324 transsternal thymectomies. *Annals of Thoracic Surgery*.

[9] Shelly S., Agmon-Levin N., Altman A., Shoenfeld Y. (2011). Thymoma and autoimmunity. *Cellular and Molecular Immunology*.

[10] Lindner A., Schalke B., Toyka K. V. (1997). Outcome in juvenile-onset myasthenia gravis: a retrospective study with long-term follow-up of 79 patients. *Journal of Neurology*.

[11] Beekman R., Kuks J. B. M., Oosterhuis H. J. G. H. (1997). Myasthenia gravis: diagnosis and follow-up of 100 consecutive patients. *Journal of Neurology*.

[12] Amato A. A., Russell J. A., Amato A. A., Russell J. A. (2008). Disorders of neuromuscular transmission. *Neuromuscular Disorders*.

[13] Wu Y., Chen Y., Liu H., Zou S. (2015). Risk factors for developing postthymectomy myasthenic crisis in Thymoma Patients. *Journal of Cancer Research and Therapeutics*.

[14] Darnton S. J., Zgainski B., Grenier I. (1999). The use of an anabolic steroid (nandrolone decanoate) to improve nutritional status after esophageal resection for carcinoma. *Diseases of the Esophagus*.

[15] Aiko S., Yoshizumi Y., Sugiura Y. (2001). Beneficial effects of immediate enteral nutrition after esophageal cancer surgery. *Surgery Today*.

[16] Kano M., Nabeya Y., Akutsu Y., Shuto K., Uesato M., Miyazawa Y. (2009). Effect of practical use of preoperative immunonutrition with impact on prevention of postoperative pneumonia after esophagectomy. *Gan To Kagaku Ryoho*.

[17] Marano L., Porfidia R., Pezzella M. (2013). Clinical and immunological impact of early postoperative enteral immunonutrition after total gastrectomy in gastric cancer patients: a prospective randomized study. *Annals of Surgical Oncology*.

[18] Giger U., Büchler M., Farhadi J. (2007). Preoperative immunonutrition suppresses perioperative inflammatory response in patients with major abdominal surgery-a randomized controlled pilot study. *Annals of Surgical Oncology*.

[19] Correia M. I. T. D., Waitzberg D. L. (2003). The impact of malnutrition on morbidity, mortality, length of hospital stay and costs evaluated through a multivariate model analysis. *Clinical Nutrition*.

[20] Gianotti L., Braga M., Nespoli L., Radaelli G., Beneduce A., Di Carlo V. (2002). A randomized controlled trial of preoperative oral supplementation with a specialized diet in patients with gastrointestinal cancer. *Gastroenterology*.

[21] Gajdos P., Chevret S., Toyka K. V. (2012). Intravenous immunoglobulin for myasthenia gravis. *Cochrane Database of Systematic Reviews*.

[22] Eftimov F., Winer J. B., Vermeulen M., de Haan R., van Schaik I. N. (2013). Intravenous immunoglobulin for chronic inflammatory demyelinating polyradiculoneuropathy. *The Cochrane Database of Systematic Reviews*.

[23] Phillips L. H. (2003). The epidemiology of myasthenia gravis. *Annals of the New York Academy of Sciences*.

[24] Panda S., Goyal V., Behari M., Singh S., Srivastava T. (2004). Myasthenic crisis: A Retrospective Study. *Neurology India*.

[25] Fink M. E., Ropper A. H. (2016). Treatment of the critically ill patient with myasthenia gravis. *Neurological and Neurosurgical Intensive Care*.

[26] Soleimani A., Moayyeri A., Akhondzadeh S., Sadatsafavi M., Shalmani H. T., Soltanzadeh A. (2004). Frequency of myasthenic crisis in relation to thymectomy in generalized myasthenia gravis: a 17-year experience. *BMC Neurology*.

[27] Faist E., Kupper T. S., Baker C. C., Chaudry I. H., Dwyer J., Baue A. E. (1986). Depression of cellular immunity after major injury. Its association with posttraumatic complications and its reversal with immunomodulation. *Archives of Surgery*.

[28] Braga M., Gianotti L., Vignali A., Carlo V. D. (2002). Preoperative oral arginine and n-3 fatty acid supplementation improves the immunometabolic host response and outcome after colorectal resection for cancer. *Surgery*.

[29] Braga M., Gianotti L., Vignali A., Di Carlo V. (1998). Immunonutrition in gastric cancer surgical patients. *Nutrition*.

[30] Tang Y., Li J., Li F. (2015). Autophagy protects intestinal epithelial cells against Deoxynivalenol toxicity by alleviating oxidative stress via IKK signaling pathway. *Free Radical Biology and Medicine*.

[31] Sivaprakasam S., Gurav A., Paschall A. V. (2016). An essential role of Ffar2 (Gpr43) in dietary fibre-mediated promotion of healthy composition of gut microbiota and suppression of intestinal carcinogenesis. *Oncogenesis*.

[32] Rowan N. R., Johnson J. T., Fratangelo C. E., Smith B. K., Kemerer P. A., Ferris R. L. (2016). Utility of a perioperative nutritional intervention on postoperative outcomes in high-risk head & neck cancer patients. *Oral Oncology*.

[33] Moya P., Soriano-Irigaray L., Ramirez J. M. (2016). Perioperative standard oral nutrition supplements versus immunonutrition in patients undergoing colorectal resection in an enhanced recovery (ERAS) protocol. *Medicine*.

[34] Silvestri S., Franchello A., Deiro G. (2016). Preoperative oral immunonutrition versus standard preoperative oral diet in well nourished patients undergoing pancreaticoduodenectomy. *International Journal of Surgery*.

[35] Mariette C. (2015). Immunonutrition. *Journal of Visceral Surgery*.

[36] Song G. M., Tian X., Liang H. (2015). Role of enteral immunonutrition in patients undergoing surgery for gastric cancer: a systematic review and meta-analysis of randomized controlled trials. *Medicine*.

[37] Rabinstein A. A., Mueller-Kronast N. (2005). Risk of extubation failure in patients with myasthenic crisis. *Neurocritical Care*.

[38] Shimogawa T., Morioka T., Hagiwara N., Akiyama T., Sayama T., Haga S. (2015). Infection control effect of dietary fluid with whey peptide in the management of patients with severe intracranial hemorrhage during the acute stage. *Fukuoka Igaku Zasshi*.

[39] Beloribi-Djefaflia S., Vasseur S., Guillaumond F. (2016). Lipid metabolic reprogramming in cancer cells. *Oncogenesis*.

[40] Calder P. C. (2010). Fatty acids and inflammation from the membrane to thenucleus and from the laboratory bench to the clinic. *Clinical Nutrition*.

[41] Tossou M. C., Liu H., Bai M. (2016). Effect of high dietary tryptophan on intestinal morphology and tight junction protein of weaned pig. *BioMed Research International*.

[42] Gstraunthaler G., Landauer F., Pfaller W. (1992). Ammoniagenesis in LLC-PK1 cultures: role of transamination. *American Journal of Physiology-Cell Physiology*.

[43] Wischmeyer P. E. (2007). Glutamine: mode of action in critical illness. *Critical Care Medicine*.

[44] Rhoads J. M., Argenzio R. A., Chen W. (1997). L-glutamine stimulates intestinal cell proliferation and activates mitogen-activated protein kinases. *American Journal of Physiology—Gastrointestinal and Liver Physiology*.

[45] Barbul A., Lazarou S. A., Efron D. T., Wasserkrug H. L., Efron G. (1990). Arginine enhances wound healing and lymphocyte immune responses in humans. *Surgery*.

[46] Gunerhan Y., Koksal N., Sahin U. Y., Uzun M. A., Ekşioglu-Demiralp E. (2009). Effect of preoperative immunonutrition and other nutrition models on cellular immune parameters. *World Journal of Gastroenterology*.

[47] Xu J., Zhong Y., Jing D., Wu Z. (2006). Preoperative enteral immunonutrition improves postoperative outcome in patients with gastrointestinal cancer. *World Journal of Surgery*.

